# Factors Influencing the Adoption of Health Information Standards in Health Care Organizations: A Systematic Review Based on Best Fit Framework Synthesis

**DOI:** 10.2196/17334

**Published:** 2020-05-15

**Authors:** Lu Han, Jing Liu, Richard Evans, Yang Song, Jingdong Ma

**Affiliations:** 1 School of Medicine and Health Management Tongji Medical College Huazhong University of Science and Technology Wuhan China; 2 College of Engineering Design and Physical Sciences Brunel University London London United Kingdom

**Keywords:** health information systems, health information interoperability, adoption, health care sector

## Abstract

**Background:**

Since the early 1970s, health care provision has experienced rapid growth in the investment and adoption of health information technologies (HITs). However, the development and deployment of HITs has often been conducted in silos, at different organizational levels, within different regions, and in various health care settings; this has resulted in their infrastructures often being difficult to manage or integrate. Health information standards (ie, the set norms and requirements that underpin the deployment of HITs in health care settings) are expected to address these issues, yet their adoption remains to be frustratingly low among health care information technology vendors.

**Objective:**

This study aimed to synthesize a comprehensive framework of factors that affect the adoption and deployment of health information standards by health care organizations.

**Methods:**

First, electronic databases, including Web of Science, Scopus, and PubMed, were searched for relevant articles, with the results being exported to the EndNote reference management software. Second, study selection was conducted according to pre-established inclusion and exclusion criteria. Finally, a synthesized best fit framework was created, which integrated a thematic analysis of the included articles.

**Results:**

In total, 35 records were incorporated into the synthesized framework, with 4 dimensions being identified: technology, organization, environment, and interorganizational relationships. The technology dimension included relative advantage, complexity, compatibility, trialability, observability, switching cost, standards uncertainty, and shared business process attributes. The organization dimension included organizational scale, organizational culture, staff resistance to change, staff training, top management support, and organizational readiness. The environment dimension included external pressure, external support, network externality, installed base, and information communication. Finally, the interorganizational relationships dimension included partner trust, partner dependence, relationship commitment, and partner power.

**Conclusions:**

The synthesized framework presented in this paper extends the current understanding of the factors that influence the adoption of health information standards in health care organizations. It provides policy and decision makers with a greater awareness of factors that hinder or facilitate their adoption, enabling better judgement and development of adoption intervention strategies. Furthermore, suggestions for future research are provided.

## Introduction

### Background

During the last 50 years, the health care sector has experienced rapid technological growth, with the investment and adoption of health information technologies (HITs) showing promise to increase patient safety, reduce medical errors, improve efficiency, and reduce overall costs. However, health care systems are inherently complex, incorporating numerous interrelated and independent components [1]. A plethora of HITs exist across different levels of health care organizations [2]; however, underlying infrastructural issues have caused a multitude of integration and management issues [3]. This has resulted in many limitations, resulting in organizations not reaping the adoption benefits that were once promised, in particular, reduction in medical service costs [4]. For this reason, HITs should be adopted in a way that creates interoperability with other health care systems, enabling organizations to realize such benefits [5]. This can be resolved through the implementation of consensus standards [6]. The use of consensus standards is based on the idea of developing an agreed set of specifications or standards for data exchange that are not dependent on any proprietary software and are universally understood and accepted for data exchange [7].

### Objective

Despite health information standards being seen as fundamental to the development of interoperable solutions [8,9], their adoption remains to be frustratingly low among information technology (IT) vendors and health organizations [10]. Prior studies have shown that the adoption of such standards in health care organizations is scarce [11-13]; however, there has been some exploration into the adoption of information standards not just in the health care sector. According to the results of these studies, different adoption factors may lead to difficulties for decision makers to explicitly understand, measure, and decrease inhibiting factors or enhance facilitating forces [14]. Hence, there is a need to synthesize those insights to provide decision makers with a holistic view of the adoption of health information standards. To achieve this goal and bridge the research divide, a comprehensive framework of factors that influence the adoption of health information standards is synthesized in this paper. The synthesized framework provides policy and decision makers with a more informed understanding of the factors that hinder or facilitate the adoption of health information standards, enabling better judgement and development of suitable strategies for adoption intervention. The following research questions (RQs) were proposed in this study：

- RQ1: What common factors have been included in previous studies that influence the adoption of health information standards by health care organizations?
- RQ2: Is there a framework that contains these factors more comprehensively from different dimensions?
- RQ3: If so, how will the adoption factors, included in the presented comprehensive framework, specifically affect the adoption of health information standards by health care organizations?

To answer these questions, this study aimed to identify and review existing articles on the adoption of information standards, extracting and summarizing their adoption factors to create a synthesized framework of the factors that affect the adoption of health information standards by health care organizations. A substantial number of stakeholders, including policy makers, citizens/patients, health care IT vendors, health care business owners, assessment bodies and regulators, clinicians and health care professionals, authorities and public administration departments, funders and health insurance companies, and academic departments, will find the presented framework beneficial in practice and when considering future research directions.

## Methods

### Study Design

A systematic review and framework synthesis were used as the methodological underpinning for our study. The systematic review was conducted according to the Preferred Reporting Items for Systematic Reviews and Meta-Analyses (PRISMA) [15], whereas the *best fit* framework synthesis approach, proposed by Booth and Carroll [16], was adopted. The *best fit* approach is a relatively recent development, adapted from framework analysis, which involves systematically organizing data into an a priori conceptual framework. This study employed this approach for 2 reasons. First, the technology-organization-environment (TOE) framework, proposed by Tornatzky and Fleischer [17], seen as the most suitable framework for understanding technology adoption in organizational contexts, can be used as an a priori framework to integrate the factors that influence the adoption of health information standards. Second, although the approach is largely deductive (testing a framework), it also includes an inductive (thematic) analysis that is useful in understanding the phenomenon, especially the adoption of information standards from a health care perspective. Thus, this study will use the *best fit* approach to synthesize a comprehensive framework of factors affecting the adoption of health information standards by organizations based on the retrieved literature.

### Search Strategy

This study comprehensively searched for all relevant literature in 3 electronic databases: Web of Science, Scopus, and PubMed. The search strategy employed is described in the following sections.

#### Web of Science

The Web of Science database was searched on July 25, 2019, and included 216 documents. The keywords used were as follows:

TS=(“information” OR “data”) AND TI=(“standards”) AND TI=(“adopt*” OR “accept*” OR “implement*”) AND TS=(“factors” OR “determinants” OR “barriers” OR ”facilitators”)

LANGUAGE=English

#### Scopus

The Scopus database was searched on July 25, 2019, and included 209 documents. The keywords used were as follows:

(TITLE-ABS-KEY(“information” OR “data”) AND TITLE(“standards”) AND TITLE(“adopt*” OR “accept*” OR “implement*”) AND TITLE-ABS-KEY(“factors” OR “determinants” OR “barriers” OR ”facilitators”))AND (LIMIT-TO(LANGUAGE, “English”))

#### PubMed

The PubMed database was searched on July 25, 2019, and included 36 documents. The keywords used were as follows:

((((information OR data)) AND standards[Title]) AND (adopt*[Title] OR accept*[Title] OR implement*[Title])) AND (factors OR determinants OR barriers OR facilitators). Filters: English

### Inclusion Criteria

Studies were considered eligible (1) if they were related to the adoption of protocol, data sets, classification, coding, specification, terminology, identification, system framework, assessment, and other information or data standards; (2) if they involved research into the factors (including barriers and facilitators) that influence the adoption or implementation of standards; and (3) if they were based on relevant adoption theories, models, or frameworks, or if they involved the proposal of an adoption model or framework.

### Exclusion Criteria

Studies considered ineligible for this research included those that (1) were not focused on the adoption of information or data standards; (2) did not involve factors that influenced standard adoption; or (3) did not involve relevant adoption theories, models, or frameworks.

### Study Selection

In this study, search results were exported and indexed in EndNote X9.2, a reference management software. Once duplicates and patent documents were removed, LH screened the titles and abstracts of all remaining records for relevance. In the next step, the full-text articles of the retrieved results were examined by LH and JL for inclusion. Discrepancies were adjudicated by a senior researcher (JM).

### Data Extraction and Synthesis

In this study, the *best fit* framework synthesis approach was followed, which integrates a thematic analysis to synthesize a comprehensive framework. The process consisted of the following stages:

1. *Familiarization with collected data.* On the basis of the understanding of the terminologies or terms used in the included studies, the factors influencing the adoption of information standards were initially extracted.
2. *Generation of initial codes.* According to definitions used in the identified studies, the extracted adoption factors were examined successively to make necessary mergers and trade-offs, generating a list of factors appropriate for health information standard adoption scenarios. The process included the following situations: (1) the factors with the same or similar meanings were combined into the same one and named with the most common term used in the literature; (2) the factors with different meanings were considered as juxtaposed dissimilar ones; and (3) if one factor was subordinate to another, the former was subsumed into the latter. For instance, *expected benefits* had the same meaning as *relative advantage*; these were combined into the same factor and named the latter. Similarly, *government support, vendor support,* and *partner support* were all related to *external support*, with the first three being subsumed into the last.
3. *Search for themes and define and name themes.* This stage consisted of 2 steps. First, the 3 dimensions of the prior framework (TOE) were used as initial themes for a deductive analysis, that is, based on the perceived commonality of the themes, the factors were analyzed and organized into 3 dimensions: technology, organization, and environment. The TOE framework explains that an organization’s decision to adopt technology can be jointly explained by 3 comprehensive dimensions, including technological, organizational, and environmental contexts. The technological context is essentially described by depicting the important attributes of the technology. The organizational context is depicted using descriptive measures concerning the organization (eg, scope, size, and managerial structure) and is influenced by formal and informal intraorganizational mechanisms for communication and control. The resources and innovativeness of the organization also play a role. The environmental context refers to the different attributes of the external environment in which an organization operates [18]. In the second step, apart from the 3 dimensions, another cluster of adoption factors, which could not be mapped against the TOE framework, was identified. The factors in this cluster were subsequently inductively analyzed, and a new dimension, titled interorganizational relationships, was generated. The interorganizational relationships are concerned with relationships between and among organizations, and it is a complex concept including many aspects, such as partner uncertainty, power, trust, and intermediary of relationship.
4. *Review themes.* This stage consisted of 2 levels. First, reviewing at the level of coded data. All adoption factors were reanalyzed within and across the dimensions to ensure consistency and independence. Second, reviewing at the level of themes. The dimensions were reviewed one final time to ensure they reflected the meaning of the adoption factors.

Ultimately, a comprehensive framework containing 4 dimensions (ie, technology, organization, environment, and interorganizational relationships) was synthesized. Throughout the synthesis, to ensure consistency in the classification of adoption factors, 3 researchers (LH, JL, and JM) discussed the factors to eliminate divergence.

## Results

### Search Results

**Figure 1:**
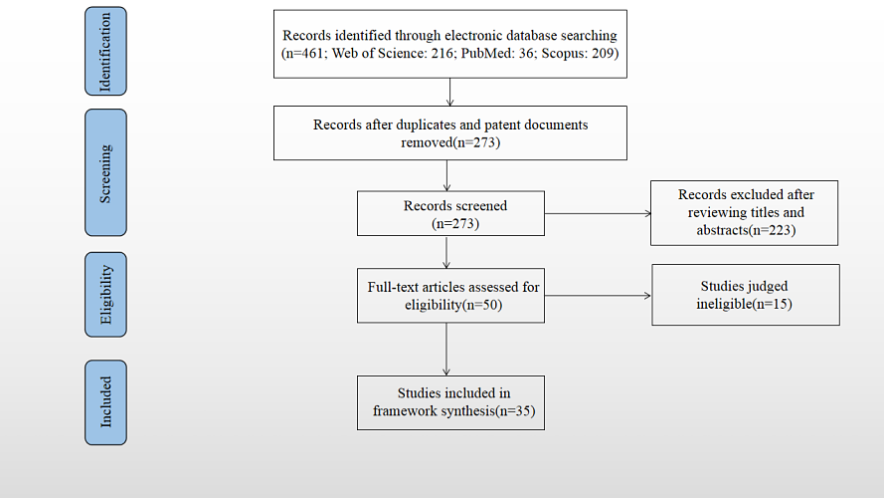
The Preferred Reporting Items for Systematic Reviews and Meta-Analyses flow diagram.

In this study, 461 records were retrieved from the ISI Web of Science, Scopus, and PubMed databases. After removing 162 duplicate and 26 patent documents, the remaining 273 records were screened based on their titles and abstracts, according to the inclusion and exclusion criteria. As a result, 223 articles were deemed ineligible and were excluded. Then, after examining the full texts of the remaining 50 articles, 35 met the inclusion criteria and were included in the final review. Articles were excluded for the following reasons: 2 studies did not focus on the adoption of information or data standards; 2 studies did not involve factors that influence adoption; 2 studies were presented without relevant adoption theories, models, or frameworks; and 9 studies were not available in full. A flowchart summary of the literature search conducted is presented in the PRISMA diagram shown in Figure 1.

### Characteristics of Included Studies

The 35 articles included in this synthesis were mainly published from 2010 to 2018 (24/35, 68%). Among the included studies, 19 employed a quantitative design, 15 were qualitative, and 1 adopted a mixed methods approach. The quantitative studies mainly employed a questionnaire or survey, whereas the qualitative studies largely used interviews and focus group discussions. Eight studies were related to the adoption of information standards in the medical field, such as Health Level seven [13], health data standards [12,19,20], and data protection standards [21]. The remaining 27 focused on the IT field. For example, Internet Protocol version 6 [22-24], RosettaNet [25-27], and electronic data interchange [28-30]. Only 13 articles comprehensively considered the 3 dimensions of technology, organization, and environment [12,13,18,22,25,26,28,30-35], whereas one of them also included interorganizational determinants [28].

### Results of Synthesis

**Figure 2:**
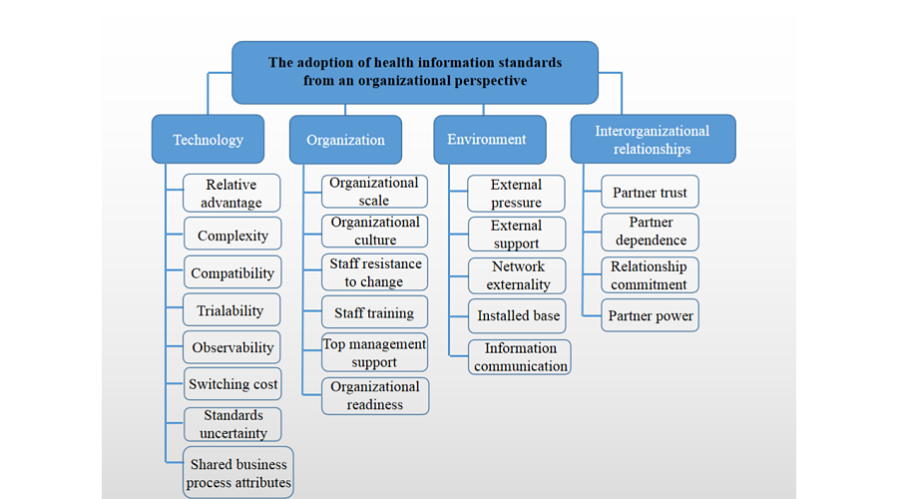
The synthesized framework.

This study took the adopting organization as the unit of analysis. On the basis of the *best fit* framework synthesis approach, the final synthesized framework included technology, organization, environment, and interorganizational relationships (Figure 2). The technology dimension incorporated relative advantage, complexity, compatibility, trialability, observability, switching cost, standards uncertainty, and shared business process attributes. The organization dimension included organizational scale, organizational culture, staff resistance to change, staff training, top management support, and organizational readiness. The environment dimension contained external pressure, external support, network externality, installed base, and information communication. Finally, the interorganizational relationships dimension included partner trust, partner dependence, relationship commitment, and partner power. These common factors identified in the included studies will have an impact on the adoption of health information standards by health care organizations. The specific impact of these factors will be detailed in the next section. The factors that influence the adoption of health information standards under the 4 dimensions are shown in Table 1 (for the definition of each factor, see Multimedia Appendix 1).

**Table 1:** Factors that influence the adoption of health information standards under the 4 dimensions.

Dimensions and factors		References
**Technology**		
	Relative advantage	[22,24,26,28,30-41]
	Complexity	[12,13,22,24,26,28,31-34,36,38,41,42]
	Compatibility	[12,13,18,24-26,28,32-36,38,41,42]
	Observability	[24,26,41,42]
	Trialability	[24,26,34,36,41,43]
	Switching cost	[12,23,34,44,45]
	Standards uncertainty	[25]
	Shared business process attributes	[35]
**Organization**		
	Organizational scale	[13,26,28,31,32,38,41]
	Organizational culture	[12,25,26,31,41,46]
	Staff resistance to change	[12]
	Staff training	[18,46]
	Top management support	[18,22,26,28,32,35,37,38,40,42,46]
	Organizational readiness	[26,28,30-32,34,35,38,40,41,43,44,47,48]
**Environment**		
	External pressure	[18,22,23,27,30,37,43,48-50]
	External support	[13,18,23,24,26,27,32,34,38,40,43,46,49]
	Network externality	[12,24,28,34,44,45]
	Installed base	[24,31,33]
	Information communication	[24,50]
**Interorganizational relationships**		
	Partner trust	[26-29,43]
	Partner dependence	[28,29]
	Relationship commitment	[26,28,29]
	Partner power	[26,27]

## Discussion

### Principal Findings

As identified in the included studies, there exist various objects (standards), fields of inquiry, and methodological approaches when it comes to exploring factors that influence the adoption of health information standards; each study has its own specific object and approach (for details of included studies, see Multimedia Appendix 2). In all studies, the adoption factors were identified and selected according to relevant theories, models, or frameworks and the specific standard. The resulting differences may be partly because of the different characteristics of the adopted standards and their different requirements for the adoption environment. However, the factors that influence their adoption can be useful in better understanding the adoption of health information standards by organizations. This study sought to identify the contributing factors that influence the adoption of health information standards in the health care sector, providing a comprehensive synthesized framework. As previously mentioned, the adoption factors have been organized into 4 dimensions, as explained in the following sections.

#### Technology Dimension

The characteristics of innovation have been frequently studied in research relating to innovation adoption. Whether an innovation can be adopted by an individual, organization, or industry and its own characteristics and advantages, namely, its own technical factors, play a pivotal role. Therefore, the technical factors of the adopted standards are the primary consideration for the adoption of health information standards. The results of this study indicate that 19 studies used factors of technical characteristics for assessing the impact on adopting information standards [12,13,18,22-26,28,30-43,45]. In this study, relative advantage, complexity, compatibility, trialability, observability, switching cost, standards uncertainty, and shared business process attributes were included in the synthesized framework.

Roger identified 5 perceived attributes of an innovation that may determine the innovation’s rate of adoption [51]. These attributes are relative advantage, complexity, compatibility, trialability, and observability, which are deemed useful for assessing the decision to adopt standards. The degree of relative advantage may be measured in economic terms, such as faster development, less maintenance, and cost saving [41]; these advantages could generate new markets, products, and services, which in turn create a competitive advantage for early adopters [24]. Thus, the greater the perceived relative advantage of the standard, the more rapid its rate of adoption will be [41]. The increased complexity of each standard increases the effort required to implement it and, therefore, reduces the number of potential adopters [24]. Thus, the more complex a standard, the less likely it is to be adopted by the organization. If the adopted standard is compatible with existing technologies or infrastructure, and consistent with past experiences of the organization, the organization will tend to upgrade to the new standard to gain a competitive advantage. Furthermore, if the adopted standard is of high trialability, the organization can reduce uncertainty and the risk of deploying the standard and obtain an increased perceived value through an initial pilot study, which will increase the organization’s willingness to adopt the standard. Similarly, if the adopted standard has significant observable benefits and quantifiable advantages, it will reduce the perceived risk and make the organization more willing to adopt the standard. In summary, when the adoption of standards is perceived as having greater relative advantage, compatibility, trialability, observability, and less complexity, the organization will be more inclined to adopt standards [24,26,41].

The cost of switching between standards was observed as a negative factor to standard adoption by health care organizations [12,23,34]. Cost is typically associated with the unfamiliarity of the organization with existing resources and skills regarding the standards. For example, if there is a lack of experts who can deal with or lead the adoption, then it will cost large sums to consult relevant experts. As a result, a great deal of staff training, and a high degree of change management, will be required. Mapping issues from the old information infrastructure to the new standardized one will also be a real cost concern; thus, the organization will consider that it has already invested in their current infrastructure and will be reluctant to discard an amount of capital and equipment, as a result of the requirements of adopting the new standard [12].

Standards uncertainty represents the perception of whether the process specifications and associated technologies will be stable, over a certain period, and able to deliver the intended benefits [25]. As David and Greenstein [52] noted, a firm may not be willing to adopt a standard until it becomes a de facto standard in the industry. Thus, if decision makers perceive that the technology and processes required for standard adoption are not stable and are not going to change in the future, this will hinder the adoption of standards by organizations. Finally, as adopted standards are often based on business processes and information sharing between organizations, shared business process attributes, such as transaction volume needs, timeliness of exchange, effectiveness of communications, accuracy and integrity needs, and collaboration levels between participants, will influence the organization’s decision on whether to adopt the standards or not [35].

#### Organization Dimension

Choosing whether to adopt standards or not is an organizational-level decision executed in an interorganizational context. There are various aspects of standard adoption that cannot be explained by technical factors alone [28]. Although the adoption of health information standards will promote better information sharing and connectivity within and between organizations, there are certain risks and uncertainties in adoption behavior because of past experiences; hence, organizational factors play a significant role in decision making. On the basis of our findings, 19 studies used organizational characteristic factors to assess the impact of adopting information standards [12,13,18,22,25,26,28,30-35,37,38, 40-43,46-48]. In this study, organizational scale, organizational culture, staff resistance to change, staff training, top management support, and organizational readiness were included in the synthesized framework.

Organizational size makes a significant contribution to the adoption of standards [13,26,28,31,32,38,41]. According to some prior studies, large enterprises have several advantages over smaller ones. Large enterprises command considerable funds, talent, and research and development capacity, so they can realize the envisaged benefits quickly after adoption. On the contrary, other studies suggest that the bureaucracy of large enterprises is more complex and requires more time for decision making. Small- and medium-sized enterprises are effective and more conducive to adopting new technologies because of their efficient top-down introduction process; however, examination of the introduction effect may require further analysis to determine this conclusion.

Organizations that have a culture of innovation are more likely to experiment with standards at earlier stages [41]. Similarly, organizations should seek to strengthen internal knowledge management practices by constructing a learning organization, as knowledge management enables the knowledge of employees to evolve into the knowledge of the organization and teams. Organizations with rich knowledge of standard adoption are more likely to make decisions quicker and more effectively [31]. Furthermore, an organization’s willingness to share information with its trading partners plays a key role in the success of standard adoption [26]. In short, organizations with a culture of innovation, learning, and information sharing are more likely to be early adopters of standards [12,25,26,31,41,46].

Alkraiji et al [12] established that employee reactions are a barrier to the adoption of health information standards because of the lack of understanding of the importance and benefits brought by standards. In addition, the staff’s resistance to change also comes from their lack of relevant technical knowledge and ability. Sobol et al [53] indicated that the IT knowledge and capabilities of employees critically influence medical computerized system implementation; in other words, if the staff were more knowledgeable about standards, there would be fewer advocator obstacles and less resistance against adoption.

Training is also deemed an important organizational mechanism that contributes to implementation success [54,55]. Employees must acquire new knowledge based on the understanding of the need for change to be able to overcome knowledge barriers and thus adopt new innovations effectively. Having an adequate training program is likely to increase employees’ confidence and reduce resistance to standard adoption [56]. Moreover, training has been proven to enhance employee productivity and assist in utilizing the innovation to its full potential, which in turn can help organizations realize the full benefits derived from an innovation [57,58]. Therefore, the development of staff training effectively improves employees’ relevant skills, capabilities, and knowledge of standard adoption, thus promoting the adoption process [18,46].

Previous studies have shown that top management support has a positive effect on technology adoption [13,59,60]. Senior managers can provide a long-term strategic vision, initiatives, and commitment to create a positive environment suitable for change [61]. Top management support can also enhance work satisfaction by modifying the rules and procedures that regulate and motivate employees’ behavior to overcome the resistance to innovation implementation [62]. Young and Poon [63] asserted that top-level management support is essential in promoting interest and employees’ satisfaction with the innovation. In the context of standard adoption, a high level of top management support means that top managers understand the benefits associated with the standard and demonstrate their commitment and political support. Therefore, top management support is expected to have a positive effect on standard adoption [18,22,26,28,32,35,38,42,46].

The success of innovation adoption is further dependent on an organization’s preparation for the innovation. Organizational readiness, including technology readiness and resource readiness, can be used to measure an organization’s capabilities for innovation adoption. Technology readiness refers to the level of sophistication of IT usage and management in an organization [35]. It includes top-level support from managers for related technologies [64], IT personnel, professional knowledge, skills, and experiences required for standard adoption [28,41]. Resource readiness measures whether an organization has enough resources to undertake the adoption [65]. It includes available financial resources to pay for installation costs, implementation of any subsequent enhancements, and ongoing expenses during usage [35], as well as other necessary resources, such as human resources, material resources, and information resources. If an organization has a high level of technology and resource readiness, it will have sufficient capacity to adopt standards, which will enable the organization to make decisions on standard adoption [26,28,30-32,34,35,38,41,43,48].

#### Environmental Dimension

All organizations exist in a certain social environment and will inevitably be affected by various external factors. When it comes to the adoption of health information standards, environment is a force that can encourage or impede an organization to adopt standards [28]; thus, environmental factors are also important factors that cannot be ignored. On the basis of the data extracted from the literature, 23 studies used environmental characteristic factors to assess the impact of adopting information standards [12,13,18,22-28,30-35,37,38,40,42,43,45,46,48-50]. In this study, external pressure, external support, network externality, installed base, and information communication were included in the synthesized framework.

An organization’s decision to adopt standards is stimulated by pressures from various external sources, including the government [66-68], the industry in which it operates (ie, business partners and/or competitors) [69-71], and other sources, such as suppliers, customers, regulatory agencies, and professional associations [18]. Under the stimulation of these pressures, organizations may adopt relevant standards to seek sustainable development or actively strive for market competitiveness [18,22,23,27,30,43,48-50].

The level of external support is critical to the adoption of standards [13,18,23,24,26,27,32,34,38,43,46,49]. Morison [72] concluded that it is difficult for an organization to adopt a new standard without the intervention of an external agent in a position of power. Here, external support includes that from the government [30,73-75], which refers to governmental support for standard adoption through financial incentives, tax cuts, and pilot programs [49] and other forms of support that come from suppliers, external experts, and consultants [18,38,49], which will provide the organization with the necessary assistance and impetus to adopt standards.

Network externalities is one of the 2 main theories used within the stream of an economics perspective of standards and is related to the benefits created through the adoption of new standards by the potential community of adopters [12]. Positive network externalities provide support to the expectations of widespread adoption of a standard. Typically, the result is a reduction in cost because of the economies of scale and synergies created through increased opportunities of interactions among adopters [24]. As more organizations adopt the standard, barriers to adoption for others in the community are lowered [76,77]; thus, the network externalities have a positive influence on organizations to adopt standards [12,24,28,34].

In an internet environment that emphasizes interoperability, the large existing installed base and the resulting inertia (perception of switching costs and sunk costs) have a significant negative impact on the adoption of standards by organizations [24,31,33]. Farrell and Saloner [78] suggested that the current state of infrastructure, characterized by its installed base, the resulting inertia, and sunk costs in existing technology, can play an important role in determining the attractiveness of the environment for adoption. A well-established standard with a large installed base can create high drag and inertia, making the environment less attractive, thereby deterring organizations from adopting the new standard [24].

For an innovation to be adopted, information about it must be available to potential adopters [79,80]. The extent of information availability will depend on the level and nature of communication within the industry [81]. An environment with successful adoption cases and pioneering adopters can provide favorable preconditions for information communication among organizations, thus raising awareness and encouraging innovation adoption [82]. Researchers view communication as vital to encourage the voluntary adoption of a new technology; this is because of a voluntary environment, where the lack of information might prompt other organizations to view the technology as risky, which fights against adoption [50]. Therefore, the effective communication of information relating to standards will play a positive role in propelling standard adoption by organizations [24,50].

#### Interorganizational Relationships Dimension

As the adoption of health information standards requires cooperation between two organizations, the relationship between an organization and its partner is salient [28]. In a technologically mature society, technology outsourcing becomes a prevalent method of satisfying an organization’s technology needs. Technical issues become relatively insignificant compared with the interorganizational relationships in information standard adoption [83,84]. The major challenge is to build new electronic relationships [70,85]. Of the 35 included studies, 5 used factors of interorganizational relationships in assessing the impact on information standard adoption, which were grouped into a new dimension in this study [26-29,43]. Partner trust, partner dependence, relationship commitment, and partner power were included in the synthesized framework.

The trust between organizations lowers stress and improves adaptability [86]. In addition, information exchange is facilitated, and the effectiveness of joint problem solving is improved [87]. According to Shang et al [88], trust was an important factor in explaining interorganizational relationships. When business partners collaborated in their supply chains, an organization that trusted its partners was more likely to reach a consensus in terms of achievable benefits by the adoption of standards [27]. Thus, partner trust facilitates the adoption of standards by organizations [26-29,43].

Interdependence results from a relationship in which both organizations perceive mutual benefits from interaction [89] and in which any loss of autonomy will be equitably compensated through the expected gains. Both parties recognize that the advantages of interdependence provide benefits greater than those that either parties could attain by themselves [90]. Therefore, the interdependence will enable the partners to rely on each other and benefit from the adoption of standards based on a high degree of cooperation, which will facilitate the adoption of standards by organizations [28,29].

Another important antecedent for promoting standard adoption includes partner commitment to the trading relationship [29]. Commitment represents the willingness of trading partners to make efforts toward the relationship. Information standards requires a richer, more cooperative relationship [91]. The standard adopters working collectively with their trading partners can provide better service to customers (or suppliers), thereby increasing their market share [29]. Hence, if the partners can take coordinated actions, based on commitment to the relationship, it will be beneficial for both parties to reach a consensus on the adoption of standards [26,28,29].

It is possible that an organization may exert pressure on its trading partners to adopt standards based on partner power [26,27]. Partner power is defined as the capability of an organization to exert an influence on another organization to act in a prescribed manner [92]. Therefore, it is possible that in an interorganizational relationship, organizations with larger partner power can use compulsory or convincing power over their business partners in the adoption of standards [93].

The aforementioned factors that influence the adoption of information standards are extracted from the retrieved literature. The careful and comprehensive consideration and categorization of these factors yield a conceptual framework that can be used as a model for the adoption of health information standards, while remaining subject to adjustment and customization according to specific health information standards and the environment in which they are adopted. The adoption of health information standards can be illustrated in this conceptual framework against 4 dimensions: technological, organizational, environmental, and interorganizational relationships. Any consideration from a single perspective could be biased and fail to provide an accurate delineation of the phenomenon. However, it is worth noting that the synthesized conceptual framework was developed based on an extensive literature review related to information standard adoption and is currently in a preliminary stage. The relationships between the 4 dimensions contained in the framework and the relationships between the adoption factors and the adoption of health information standards by health care organizations can be examined through further empirical studies.

### Limitations

This study has some limitations that should be acknowledged. First, because of the broad connotation of information standards, the search strategy employed in this study did not fully cover all concepts of information standards, which may lead to potential articles not being identified. Furthermore, because of resource constraints, the databases retrieved in this study were limited, which may result in other relevant studies not being retrieved. Second, this study excluded articles that did not involve relevant adoption theories, models, or frameworks and may have omitted some articles that solely proposed adoption factors. Finally, because of the overlap and intersection between the concepts of the adoption factors involved in the literature, there exists some subjectivity and bias in the concept definition and selection of factors and organizing the factors into corresponding dimensions in the synthesized framework.

In view of the above limitations, the synthesized framework may not include all possible adoption factors, which should be further improved and supplemented by research in the future. Nevertheless, this study has fully considered the factors that influence the adoption of health information standards, and the comprehensive framework provides references for future research and insights into the formulation and adoption of health information standards.

### Conclusions

This study has comprehensively reviewed the factors that influence the adoption of information standards in the published literature. A synthesized framework of integrated factors that influence the adoption of health information standards by organizations was extracted and presented.

This study delivers contributions at different levels. First, at the theoretical level, the synthesized framework has addressed knowledge gaps in the adoption of health information standards in health care organizations. Second, at the practice level, it will help guide policy and decision makers in better judging and developing suitable strategies for adoption interventions. For health care organizations, in particular, strategies for the adoption interventions include upgrading infrastructure and enriching technical resources and skills to better adapt to new standards; establishing an innovative culture, strengthening staff training, raising the attention of top managers, and increasing the investment of technology and resources to promote the implementation of new standards; heading on competitive pressure, leveraging external forces and information communication channels, and overcoming the industry inertia to actively respond to the adoption of new standards; and establishing trust and interdependency relationships among partners based on commitment and making reasonable use of partner power to create the industry fashion of standard adoption. Furthermore, it also provides directions for future research to enrich the factors that influence the adoption of relevant standards or health care technologies.

## Appendix

Multimedia Appendix 1 The definition of each factor.

Multimedia Appendix 2 Details of included studies.

## Abbreviations

HIT: health information technology
IT: information technology
PRISMA: Preferred Reporting Items for Systematic Reviews and Meta-Analyses
RQ: research question
TOE: technology-organization-environment
