# Supplementary material for: Factors Influencing the Adoption of Health Information Standards in Health Care Organizations: A Systematic Review Based on Best Fit Framework Synthesis
Source: JMIR Med Inform. 2020 May 15;8(5):e17334. doi: 10.2196/17334 (PMC7260665; doi:10.2196/17334)
Supplement: Multimedia Appendix 1 [file medinform_v8i5e17334_app1.docx]

**Multimedia Appendix 1**

**Table 1.** The definition of each factor.

| **Dimensions** | **Factors** | **Definition** |
| --- | --- | --- |
| Technology | Relative advantage | The degree to which the potential adopters perceive the standard as being better than that which it supersedes. |
|  | Complexity | The degree to which the standard is perceived as difficult to understand and / or adopt. |
|  | Compatibility | The degree to which the standard is perceived as being consistent with the existing technologies and past experiences of potential adopters. |
|  | Observability | The degree to which the results of standard adoption are visible to others. |
|  | Trialability | The degree to which standard adoption may be experimented with on a limited basis. |
|  | Switching cost | The added cost of an organization switching from one standard to another. |
|  | Standards uncertainty | The perception of potential adopters towards whether the process specifications and associated technologies of standard adoption will be stable over a period of time and able to deliver the intended benefits. |
|  | Shared business process attributes | The characteristics of the shared business process, such as required response times, required exchange volumes, exchange frequency, consistent field terminology and consistent business definitions, etc. |
| Organization | Organizational scale | The size of the organization. |
|  | Organizational culture | The cultural image of an organization composed of its traditions, beliefs and values. |
|  | Staff resistance to change | The restraint power of employees in the organization to oppose, obstruct or even fight against the adoption of standards. |
|  | Staff training | The organization's training activities for employees to acquire new knowledge and skills needed for the adoption of standards. |
|  | Top management support | The degree to which senior managers consider standard adoption to be important and provide the resources needed. |
|  | Organizational readiness | The organization-level attributes that assess the overall readiness of the organization towards standard adoption. |
| Environment | External pressure | Influences that arise from various sources in the competitive environment surrounding the organization. |
|  | External support | The support and sponsorship the organization receives from various external forces. |
|  | Network externality | The positive correlation between the total number of organizations in the network and the benefits each organization receives from standard adoption. |
|  | Installed base | The development and implementation basis of existing standards in the organization. |
|  | Information communication | The way in which the organization obtains relevant information on standard adoption through communication. |
| Interorganizational relationships | Partner trust | The expectation of an organization to reach a mutual respect agreement with its partners. |
|  | Partner dependence | An interdependence between an organization and its partners that benefits from their interactions. |
|  | Relationship commitment | The willingness of trading partners to make an effort towards the relationship. |
|  | Partner power | The capability of an organization to exert influence on another organization to act in a prescribed manner. |
