# Supplementary material for: Factors Influencing the Adoption of Health Information Standards in Health Care Organizations: A Systematic Review Based on Best Fit Framework Synthesis
Source: JMIR Med Inform. 2020 May 15;8(5):e17334. doi: 10.2196/17334 (PMC7260665; doi:10.2196/17334)
Supplement: Multimedia Appendix 2 [file medinform_v8i5e17334_app2.docx]

**Multimedia Appendix 2**

**Table 2.** Details of included studies.

| **Authors** | **Year** | **Object** | **Theories** | **Methods** | **Samples** | **Adoption factors** |
| --- | --- | --- | --- | --- | --- | --- |
| Gong N[18] | 2018 | CTI interoperability standards | Diffusion of innovations (DOI) | Mixed | 10 standard makers and frontier adopters in the Organization for the Advancement of Structured Information Standards (OASIS) community | Organizational readiness  External environment  Innovation attributes  Standard development organization  Network effects  Switching cost |
| Wang X & Zander S[19] | 2018 | IPV6 | Diffusion of innovation, economic perspective | Quantitative | organizations in Australia and China | Usefulness   - New feature - Relative advantage - Complexity   Environmental conditions   - Normative pressure   Organization   - Attitude toward IPV6 - Top management support - Participation |
| Van Compernolle M et al.[20] | 2018 | Data standards | Innovation Diffusion Theory (IDT), the Technology Acceptance Model (TAM), the Technology Readiness and Acceptance Model (TRAM) | Quantitative | people working in the public and private sector or as academic | Technological readiness   - Optimism - Innovativeness - Discomfort - Insecurity   Perceived ease of use  Perceived usefulness |
| Velleman E M et al.[21] | 2017 | Web accessibility standards | Diffusion of innovations model | Qualitative | 18 internal and external stakeholders who worked within municipalities during the adoption and implementation process | Adoption factors   - Relative advantage - Compatibility - Complexity or simplicity - Observability - Network externalities - Related technologies - Current infrastructure - Communication channels - Sponsorship - Resources/technical possibilities   Web design process   - Internal quality assurance - External quality assurance - Knowledge - Internal benefits - External benefits - Budget and costs   Organizational structure   - Quality of procurement - Closedness - Interdependencies - Pluralism - Managerial commitment and decisions - Responsibilities - Municipal collaboration   Personal factors   - Stakeholder influence and involvement - Disability in circle - Pride and ambition - Opinion on guidelines   External influences   - Legislation on accessibility - Other rules and demands - Citizen inflfluence |
| Ramoni R B et al.[22] | 2017 | SDDxTs | Diffusion of innovation | Quantitative | clinic deans in all US dental schools (n=61) | Compatibility   - Cultural inertia   Complexity   - Poor EHR user interface   Relative advantage   - Unclear usefulness - Not required for insurance reimbursement - Fear of a loss of autonomy - Fear of adopting a terminology that would become outmoded   Trialability   - EHR-related barriers to adoption |
| Lucho S et al.[23] | 2017 | ISO/IEC 29110 | Influencing model for Software Process Improvement(SPI) | Qualitative | 4 Very Small Entities (VSEs) | Environmental factors   - Competitive pressure - External pressure - Government support - Vendor support - Partners support - Partners readiness - Environmental Uncertainty - Vertical linkage - Partners defense - Government pressure - Number of competitors - External expertise - Consultant effectiveness - Trust with partners - Globalization - Social influence |
| Vatanasakdakul S et al.[24] | 2017 | IT governance frameworks | Technology-Organization-Environment (TOE) framework, Delone and McLean’s IS success model | Quantitative | 126 Australian organizations that have adopted IT governance frameworks | Technological Factors   - Ease of use - Innovation compatibility   Organisational Factors   - Top management Support - Training   Environmental Factors   - External support - External pressures |
| Ghahramani A[25] | 2016 | OHSAS18001 | Technology-Organization-Environment (TOE) | Qualitative | 16 managers in 3 OHSAS 18001-certifified manufacturing companies | Factors inside the organization   - Management commitment - Safety communication - Employee involvement - Integration - OHS training - Safety culture - Internal incentives   Factors outside the organization   - OHS enforcement - OHS authorities' support - Auditing - External incentives |
| Alkraiji A I et al.[12] | 2016 | Health data standards | Diffusion of innovation, Technology-Organization-Environment (TOE) | Qualitative | 6 main healthcare organizations in Saudi Arabia | Technological   - Complexity - Compatibility - Switching cost - Market uncertainties - System’s integration - Enhancing the use of advanced systems   Organisational   - Organisational characteristics - Lack of adequate policies and procedures - Resistance to change - Lack of information management plan - Data analysis - Accreditation   Environmental   - Network externalities - National healthcare system - Shortage of professionals - Lack of a national plan for medical data exchange - Lack of a national regulator |
| Mueller T et al.[26] | 2015 | Organizational IT standards | The Technology Acceptance Model (TAM), the Theory of Planned Behavior (TPB) | Qualitative | 9 interviewees with experienced IT professionals and experts from practice | Perceived behavioral control  Individual benefit  Organizational benefit  Peer group social influence  Supervisor social influence  Change of work routines |
| Azam S[27] | 2014 | SBR | Diffusion of innovation (DOI), Technology-Organization-Environment (TOE) frameworks, contingency theory | Quantitative | the top 500 companies from the ASX (Australian Securities Exchange) | Perceived environmental factors   - Competitive pressure - Government pressure - Communication in the industry |
| MacLennan E & Van Belle J P[28] | 2014 | SOA | Diffusion of innovations (DOI), institutional theory, TOE framework | Quantitative | enterprise architects in South African organizations | Use of standards and platforms  Complexity  Compatibility  Cost  Technology implementation challenges  Relative advantage  Size of an organization  Industries  Perceived risks  Organizational change implementation challenges  Top management support  SOA governance and strategy  Availability of human and financial resources  Intra-organizational benefits  Inter-organizational benefits  Vendor influence  Vendor support for integration and development tools  Industry pressure and IT media influence |
| Henning F[29] | 2013 | Interoperability standards | The innovation diffusion theory, technology acceptance theory | Qualitative | 2 Government Information Networks in the Netherlands: The Digital Client Dossier (Digitaal Klantdossier, DKD) and Studielink | IOP Governance   - Decision-Making Centralization - Enforcement - Guidance   Network Characteristics   - Network Complexity - Trust - Mimetic Dynamics - Interaction Complexity - Information Infrastructure   Network-External Environment   - Political Pressure - Policy/Institutions Support   IOP Standards Characteristics   - Customisability - Maturity - Correction Mechanisms - Trialability   Organization-Specific Determinants   - Organizational Capacity - Organizational Needs   Impacts   - Internal-Operations Outcomes - External-Relations Outcomes - Return-on-Investment Outcomes - Network-Level Outcomes - Adoption Efforts |
| Alkraiji A et al.[30] | 2013 | Health data standards | Diffusion of innovation, the perspectives of the economics of standards (network effect, switching costs) | Qualitative | 33 managers or senior officials in six tertiary healthcare organizations | Complexity  Compatibility  IT Infrastructure  Switching Cost  Market Uncertainties  Lack of Adequate Policies and Procedures  Resistance to Change  Lack of Clinicians’ Engagement  National Healthcare System  Shortage of Professionals  Lack of an Information Management Plan  Lack of a National Plan for Medical Data Exchange  Lack of a National Regulator |
| Venkatesh V & Bala H[31] | 2012 | RosettaNet-based IBPS | TOE framework | Quantitative | firms in the high-tech industry that were considering adoption of RosettaNet-based IBPS | Technological factors   - Expected benefits - Process compatibility - Standards uncertainty - Technology readiness   Organizational factors   - Organizational innovativeness   Environmental factors   - Relational trust |
| Lin C H et al.[13] | 2012 | HL7 version 2 standards | Innovation diffusion theory | Quantitative | all accredited hospitals in Taiwan | Characteristics of technology   - Complexity - Compatibility - Security   Characteristics of the organization   - Staff’s technology capability - hospital’s size   Characteristics of the environment   - Governmental promotion - Intra-industrial pressure - External support   Characteristics of top management   - Top management attitude towards IT - Top management knowledge of IT |
| Foth M et al.[32] | 2012 | Data protection standards | The Technology Acceptance Model (TAM), the Protection Motivation Theory (PMT) | Quantitative | 557 individuals of 26 hospitals in Germany | Perceived severity of data abuse  Perceived probability of data abuse  Data protection level  Perceived usefulness  Perceived ease of use  Subjective norm  Attitude |
| Chan F T S & Chong A Y L[33] | 2012 | RosettaNet | Diffusion of Innovation, TOE models, institutional theory | Quantitative | 212 Malaysian manufacturing firms | Innovation   - Compatibility - Complexity - Trialability - Relative Advantage - Observability   Organization   - Top management support - Organization size - Technical and Financial Feasibility   Environment   - Competitive pressure - Expectations of market trends   IOR   - Partner power - Relationship commitment - Trust in Trading partner - Transaction frequency   Government   - Promotion and support - Infrastructure   Information sharing culture   - Willingness to share information - Information distribution |
| Techatassanasoontorn A A & Suo S[34] | 2011 | de facto standardization | The economic theory of networks, complex network theory | Quantitative |  | Technology functionality  Adoption costs   - Price - Switching costs   Network effects |
| Hu C[35] | 2011 | Web Service Technology standards | The innovation diffusion theory, TOE(Technology, Organization, Environment) model | Qualitative |  | Influences of Technology Standards   - Advantages - Complexity - Knowledge barriers - Immature   Organizational Factors   - Technology capability of organization - Organization philosophy - Organization scale   Environmental factor   - Industry concentration - Stakeholder - Industry technical inertia |
| Hovav A et al.[36] | 2011 | IPV6 | Diffusion of innovation, the economic perspective | Quantitative | 66 Korean Internet Service Providers (ISPs) and member companies of the IPv6 Forum Korea | Usefulness for niche markets   - Killer applications - User demand   Environmental conditions   - Switching costs - Resource concentration and power - Government sponsorship - Normative pressure |
| Burbano A et al.[37] | 2011 | Identification standards | The Technology Organization and Environment (TOE) conceptual model | Qualitative | 2 experts from a local medical center; the IT VP and the supply chain manager | Technology   - Relative advantage - Compatibility - Complexity - Organization readiness(technology)   Organization   - Top management support - Hospital size - Organization readiness(process)   Environment   - Industry pressure - Vendor support - Government policy - IT vendor readiness |
| Alkraiji A et al.[38] | 2011 | Health data standards | Diffusion of innovation (DOI), the economics of standards(network effect, switching costs) | Qualitative | 8 senior managers in National Guard Health Affairs (NGHA) | Network externalities  External pressure  Integration  Data analysis  Accreditation  Standards benefits  Organization characteristics  Policy and procedure  Organization readiness  Clinician engagement  External support  Standards characteristics  Information  The immaturity of health data standards industry  Shortage of national knowledgeable experts  Lack of national plan for HIT applications and NHIN  Lack of recognized body |
| Veit D & Parasie N P[39] | 2010 | Data exchange standards | The theory of network effects, the Technology Acceptance Model (TAM), the Innovation Diffusion Theory (IDT) | Qualitative | 13 municipalities of the German federal system | External Pressure   - Pressure from Public and Private Partners - Legal Pressure   Perceived Benefits   - Economic Benefits - Political Benefits   Readiness   - Readiness of Public and Private Partners - Legal Readiness - Financial Resources - IT Sophistication |
| Huang Z et al.[40] | 2008 | Internet-EDI | Technological and organizational perspectives or economic and socio-political perspectives, Chwelos et al.’s frameworks (Technological, Organizational, Inter-organizational) | Quantitative | 658 organizations of Society for Information Management (SIM) in the mid-west region | Technological Maturity   - Relative Advantage - Compatibility - Complexity   Organizational Readiness   - Strategic Use of communication technologies - Trust in Technology - Application Knowledge - Top Management Support - Organizational Size - Organizational Slack   Environmental Pressure   - Competitive Pressure - Network Externality   Interorganizational Determinants   - Dependency on Partner - Potential Power - Exercised Power - Trust in Partner - Relationship Commitment |
| Yee-Loong Chong A & Ooi K B[41] | 2008 | RosettaNet | Diffusion of innovation (DOI) model, Technology-Organization-Environment (TOE) model | Quantitative | 400 Malaysian electrical and electronics (E&E) organizations | Trust  Products characteristics  Government’s influence  Partner’s power |
| Singh R M & Dahlin K[42] | 2007 | Technical standard | Proportional hazards model | Quantitative | the US cellular telecommunications industry | Technical merit  Concentration of ownership  Rate of increase in patent granted  The number of new patentees  Proportion of new patentees to total patentees |
| Ng C S P et al.[43] | 2006 | ERP maintenance process standard | Product standard adoption, best-practice adoption | Quantitative | ERP-client organizations in Taiwan | Vendor’s maintenance supports  Management characteristics  MIS group characteristics  Perceived benefits |
| Kelly D et al.[44] | 2006 | CNIS standards | Diffusion of innovation theory | Qualitative |  | Organizational Context   - Political Issues - Level of Support - Type and Scope of Decision Process   Standard (Innovation) Context   - Relative Advantage - Compatibility - Strategic - Technical - Interstandard - Complexity - System - Organizational Capability   External Context   - Complementary Assets - Infrastructure Investment and Installed Base - Market Uncertainty |
| Wapakabulo J et al.[45] | 2005 | STEP | Diffusion of Innovation (DOI) theory, the Economics of Standards | Qualitative | the individuals both within the Ministry of Defence (MoD) and in industry | Standard-related factors   - Complexity - Compatibility - Relative Advantage - Trialability - Stability (maturity) - Price   Organizational-related factors   - Switching costs - Resources - Champion support - User need recognition   Environmental characteristics   - Networks effects - Support infrastructure. - contractual arrangements - Sponsorship |
| Hovav A et al.[46] | 2004 | IPV6 | Diffusion of innovation, economic perspective | Qualitative |  | Usefulness of the features   - Relative advantage - Compatibility - Complexity - Trialability - Observability   Environmental Conduciveness   - Network externalities - Related technologies - Installed base/Drag - Communication channels - Sponsorship - Resources |
| Nelson M L & Shaw M J[47] | 2003 | IOS SPI | Organizational - Technological - Environmental framework | Quantitative | 102 firms from 10 different industrial groups (encompassing 15 different SDOs) | Innovation Attributes   - Relative Advantage - Compatibility - Shared business process attributes   Organizational Readiness   - Top Management Support - Feasibility - Technology Conversion   External Environment   - Competitive Pressure - Participation Level in SDO   Standards Development Organization   - Management Practices - Architecture - Governance |
| Chen M[48] | 2003 | E-business standards | The innovation diffusion model, the Technology Adoption Life Cycle | Qualitative |  | IT Standards Characteristics   - Relative advantage - Compatibility - Complexity - Trialability - Observability   Organizational Factors   - Company size & Industry type - Organizational culture - IT infrastructure - IT skill set   Stakeholders   - Customers & Suppliers - IT Product Vendors - Systems Integrators - Internal IS Organizations |
| Lee S & Lim G G[49] | 2003 | EDI | EDI implementation, inter-organizational relations and IS implementation | Quantitative | industries that have implemented EDI comprehensively and from publicly available company databases | Partnership attributes   - Partner trust - Partner interdependence - Partner commitment |
| Kuan K K Y & Chau P Y K[50] | 2001 | EDI | Technology-Organization-Environment (TOE) framework | Quantitative | 575 small firms in Hong Kong | Perceived technological benefits   - Perceived direct benefits - Perceived indirect benefits   Perceived organizational resources   - Perceived financial cost - Perceived technical competence   Perceived environmental pressure   - Perceived industry pressure - Perceived government pressure |

*CTI: Cyber Threat Intelligence

*SDDxTs: Standardized dental diagnostic terminologies

*OHSAS: The Occupational Health and Safety Assessment Series; OHS:Occupational Health and Safety

*SBR: Standard Business Reporting

*SOA: Service-oriented architecture

*IOP: Interoperability

*IBPS: Interorganizational business process standards

*HIT: Health information technology; NHIN: National health information network

*EDI: Electronic Data Interchange

*ERP: Enterprise Resource Planning

*MIS: Management Information Systems

*CNIS: Complex network-based information systems

*STEP: the Standard for the Exchange of Product Data

*IOS SPI: Interorganizational system standards and process innovations; SDO: Standards development organization
